# Mechanical Response of Glass–Epoxy Composites with Graphene Oxide Nanoparticles

**DOI:** 10.3390/ma15238545

**Published:** 2022-11-30

**Authors:** Vinayak S. Uppin, P. S. Shivakumar Gouda, M. I. Kittur, A. Andriyana, B. C. Ang, Bisma Parveez, Irfan Anjum Badruddin, Syed Javed, Sarfaraz Kamangar

**Affiliations:** 1 Department of Mechanical Engineering, Jain College of Engineering, Affiliated to Visvesvaraya Technological University, Belagavi 590014, Karnataka, India; 2 Research Center, Department of Mechanical Engineering, SDM College of Engineering and Technology, Dharwad 580002, Karnataka, India; 3 Centre of Advanced Materials, Faculty of Engineering, University of Malaya, Kuala Lumpur 50603, Malaysia; 4 Department of Mechanical Engineering, Faculty of Engineering, University of Malaya, Kuala Lumpur 50603, Malaysia; 5 Department of Chemical Engineering, Faculty of Engineering, University of Malaya, Kuala Lumpur 50603, Malaysia; 6 Department of Manufacturing and Materials Engineering, International Islamic University Malaysia, Kuala Lumpur 53100, Malaysia; 7 Mechanical Engineering Department, College of Engineering, King Khalid University, Abha 62421, Saudi Arabia

**Keywords:** mechanical properties, graphene oxide, polymer matrix composites, constitutive modeling

## Abstract

Graphene-based fillers possess exceptional properties that encourage researchers toward their incorporation in glass–epoxy (GE) polymer composites. Regarding the mechanical and wear properties of glass–epoxy composites, the effect of graphene oxide (GO) reinforced in glass–epoxy was examined. A decrease in tensile modulus and increase in tensile strength was reported for 1 wt. % of GO. A shift in glass transition temperature *T_g_* was observed with the addition of GO. The cross-link density and storage modulus of the composite decreased with the addition of GO. The decrease in dissipation energy and wear rate was reported with the increase in GO concentration. A simple one-dimensional damage model of nonlinear nature was developed to capture the stress–strain behavior of the unfilled and filled glass–epoxy composite. Tensile modulus *E*, Weibull scale parameter *σ_o_,* and Weibull shape parameter *β* were considered to develop the model. Finally, to understand the failure mechanisms in GO-filled composites, a scanning electron microscopic (SEM) examination was carried out for tensile fractured composites.

## 1. Introduction

Materials manufactured with epoxy-based content provide excellent mechanical, electrical, and thermal properties [1,2,3]. Furthermore, adding inorganic fillers to improve the properties of epoxy-based materials has become a common practice [4,5,6]. Recent studies propose that the addition of graphene oxide would be a probable reinforcement for polymer composites due to less manufacturing cost and enormously high aspect ratio leading to improved mechanical, electrical, and thermal properties [6,7,8,9,10,11,12,13,14]. The addition of 5 wt. % of graphene oxide (GO) leads to a significant enhancement in the shear strength of carbon fiber–epoxy composites [15].

The incorporation of 0.5 wt. % thermally reduced Graphene Oxide (TrGO) in the polymer matrix leads to a significant improvement in fracture strength [16]. Graphene oxide (GO) and reduced graphene oxides (rGO) are incorporated in composites to increase visible light absorption [17,18,19]. Furthermore, the addition of 2 g/m^2^ of GO results in a remarkable improvement in the fracture toughness of carbon fiber-reinforced polymer (CFRP) composite [20]. An increment of 55% is observed in residual compressive strength during the post-impact analysis of glass fiber–epoxy composites with the incorporation of 0.3% TrGO [21]. Nevertheless, an increment of only 19% is observed with the same TrGO content in carbon fiber–epoxy composites. The hydrogen barrier mechanism is improved because of GO reinforcement in the polymer composite [22]. The study of polymer composites has shown that the incorporation of fillers improves their strength, but several limitations, namely low degradation temperature, brittleness, and non-homogeneous crystallization influence the general capability of the polymer composite [22,23,24].

The inclusion of graphene nanoparticles considerably increases the composite’s tensile strength and fracture toughness. On the contrary, a significant reduction in fracture toughness is observed with the addition of a large concentration of graphene nanoparticles (greater than one) in the composite [25]. The influence of graphite as filler material on the tribological and mechanical failure in fiber-reinforced carbon-based epoxy composites was previously investigated. Mechanical properties such as shear modulus, elastic modulus, and flexural strength were enhanced with the addition of graphite in the polymer composite. In addition, sliding wear behavior was enhanced with the integration of graphite in the polymer composite [26]. The effect of graphene nano pellets on the mechanical characterization of Basalt–epoxy composites was also studied. The graphene nano pellet concentration varied from 0.1 to 0.3 wt. %. Nonetheless, the mechanical strength of the polymer composite was significantly improved with the addition of 0.1 wt. % graphene nano pellets [27].

According to an investigation of the mechanical response of glass–epoxy composite laminates covered with graphene particles, the mechanical behavior of graphene-coated fiber-reinforced composites is better than that of uncoated fibers [28]. Nevertheless, for glass–epoxy composites, comprehensive evidence on the precise quantity of graphene as filler material and its effect on tensile, dynamic, and wear behavior is subjected to discussion. With this perspective, the current study was conducted to comprehend the mechanical behavior of glass–epoxy composites with the addition of graphene oxide. Furthermore, a simple one-dimensional nonlinear mathematical model using Weibull distribution is proposed to capture the nonlinear stress–strain response of the GO–GE composite. The authors comprehend that this study will enhance the application of graphene oxide in glass–epoxy composites. In addition, the mathematical model proposed will minimize dependence on mechanical testing by approximately predicting stress with distinct graphene oxide concentrations.

## 2. Materials and Methods

### 2.1. Sample Preparation

In this investigation, individual composite laminate was fabricated with the reinforcement of a uni-directional (UD) E-glass mat of 220 GSM (grams per square meter). Commercially available Lapox L-12 resin and K-6 as a hardener/catalyst were used in the ratio of 10:1. Natural flake graphite with 85% carbon purity was utilized to synthesize graphene. Furthermore, pre-oxidation treatment was carried out using the modified Hummer method [4]. The dispersion of nanoparticles is one of the key parameters to enhance the mechanical properties. The uniform dispersion of GO without agglomeration was achieved using ultrasonication followed by a magnetic stirrer with a speed of 1500 rpm for 30 min [29]. A hardener was added during the post-stirring process. The gel time of the epoxy and hardener was 45 min. According to [30,31], with a magnetic stirrer, the agglomeration of double-layered hydroxide nanoparticles cannot be completely broken. This is because the magnetic stirrer’s input energy is insufficient to separate all the agglomerate particles into individual particles. Therefore, for easy mold release, an extended 838 mold release agent was applied to avoid surface damage.

Two different concentrations of GO, 0.5 wt. % and 1 wt. %, were mixed with epoxy using a magnetic stirrer to achieve uniform dispersion of GO in epoxy. The hand lay-up technique was adopted to manufacture the composite laminates. Initially, the mold surface was treated with a mold-releasing agent to avoid the fabricated composite sticking to the surface. A uni-directional glass mat was placed on the mold, and resin was applied over the mat. Subsequently, a hand roller was used to maintain the uniform dispersion of the matrix. Finally, the fabricated laminate was cured at room temperature for 24 h. All test samples were cut in fiber running direction (0-deg). A wheel cutter with a diamond tip was utilized at high speed to cut typical specimens from the laminates for the mechanical characterization of the composites.

### 2.2. Testing of Composites

Dynamic mechanical analysis (DMA) was conducted on TA Q-800 instruments working in three-point bending mode with a 1 Hz frequency of oscillation and ±1 modulus precision. The test was run at a 3 °C/min rate from room temperature to 165 °C. DMA tests are individual measurements with an isothermal stability of ±1 °C. The tan δ resolution and tan δ sensitivity of TA Q-800 are 0.00001 and 0.0001, respectively. Tensile tests were conducted following ASTM D3039 at room temperature, utilizing an Instron uniaxial testing machine assembled with a 400 kN load cell. The test was conducted with a crosshead speed of 2 mm/min in displacement control mode. Rectangular specimens of 250 mm × 25 mm × 4 mm were cut for the tensile test. A wear test was conducted with the aid of a pin-on-disc experimental setup with a speed of 240 rpm and 10 N load for 17 min. The dimensions of the specimens were determined according to ASTM G99, demonstrating a rectangular size of 30 mm × 10 mm × 4 mm. A set of five samples was utilized for each test.

## 3. Results and Discussion

### 3.1. Thermomechanical Analysis

**Figure 1:**
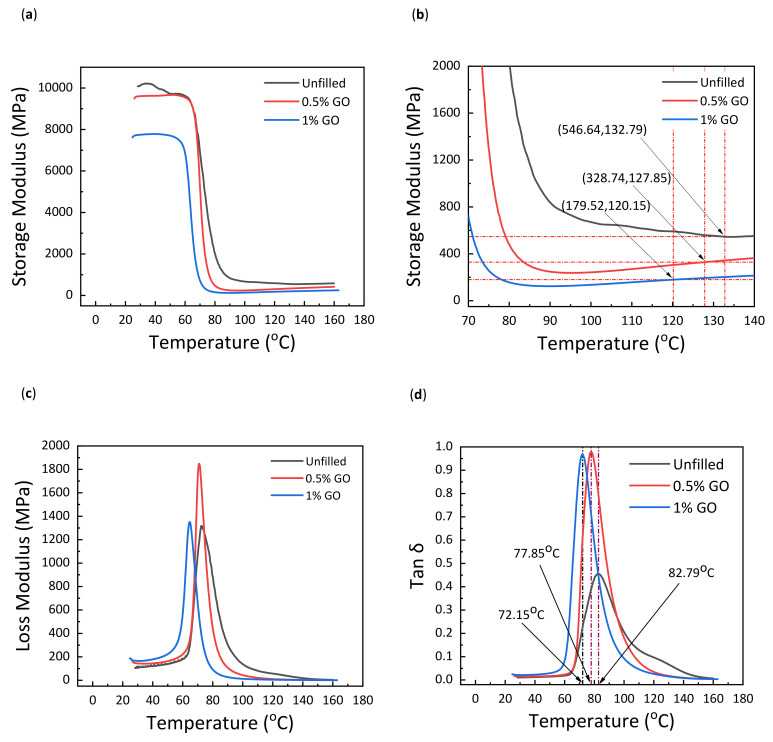
Storage modulus (**a**), storage modulus (rescaled) showing *T_g_* + 50 (**b**), loss modulus (**c**), and *tan δ* (**d**) as a function of temperature.

The influence of graphene oxide loading in glass–epoxy composites was investigated using DMA, quantifying the loss modulus, storage modulus (*G*), damping factor (*tan δ*), and glass transition temperature (*T_g_*), as shown in Figure 1. In Figure 1b, at 1% GO, the values (179.52, 120.15) correspond to the (x, y) coordinates representing the temperature and storage modulus. The loss and storage modulus values in Table 1 are listed based on experiments on the wt. % of GO in glass-epoxy composites. The sudden decrease in the value of storage modulus, which occurs around 60–70 °C for all curves, is shown in Figure 1a. This relates to the end of the glassy region for highly cross-linked thermoset polymers.

The cross-link density of the GE composite with different GO concentrations was determined using Equation (1) as (represented in Table 1):

(1) $$\rho =\frac{G}{3RT},$$

where ρ is cross-link density in mol/cm^3^, G refers to storage modulus in MPa in the rubbery plateau region, R is the universal gas constant (8.3145 J/K mol), and T is the temperature in rubbery plateau region in Kelvin at *T_g_* + 50.

As observed in Figure 1d, a declining trend in *T_g_* is reported when graphene oxide is added to the glass–epoxy matrix. A high magnitude of peak in *tan δ* is observed for 0.5 wt. % GO and 1 wt. % GO in comparison with the unfilled material. The shift in the peaks of 0.5 wt. % and 1 wt. % GO correlates with the reduction in *T_g_*. Moreover, this behavior indicates that a rise in the loss modulus shows the enhanced energy dissipation ability of the composites [32]. As shown in Figure 1c, the amalgamation of GO into GE composite causes the height of the loss modulus to rise and shift to the left. This denotes the viscoelastic effect at the GO–epoxy contact site, which results in energy dissipation [33,34].

In addition, a comparable decreasing trend was observed in the storage modulus with the addition of GO. The estimation of the corresponding cross-link density was carried out at a temperature of *T_g_* + 50, as illustrated in Figure 1b. Compared to the decrease in *T_g_*, there was a sharp reduction in cross-link density of up to 39% with the addition of 0.5 wt. % GO and 67% with 1 wt. % GO (see Table 1). Therefore, the stiffness of the polymer was strongly affected by its molecular mobility. The decrease in cross-link density increased molecular mobility, decreasing the GO–GE composite’s stiffness. Furthermore, the shift in *T_g_* was the consequence of molecular mobility modification induced by the addition of GO.

The addition of GO reduced the cross-link density and increased molecular mobility. This decrease in molecular mobility can be attributed to a decrease in *T_g_*. Higher values of cross-linking density correspond to a higher storage modulus. Thus, the highly cross-linked polymer had a much higher storage modulus demonstrating high stiffness and close-fitting network structure, whereas the polymer with lower cross-linked density demonstrated a lower storage modulus [35].

### 3.2. Tensile Test

**Figure 2:**
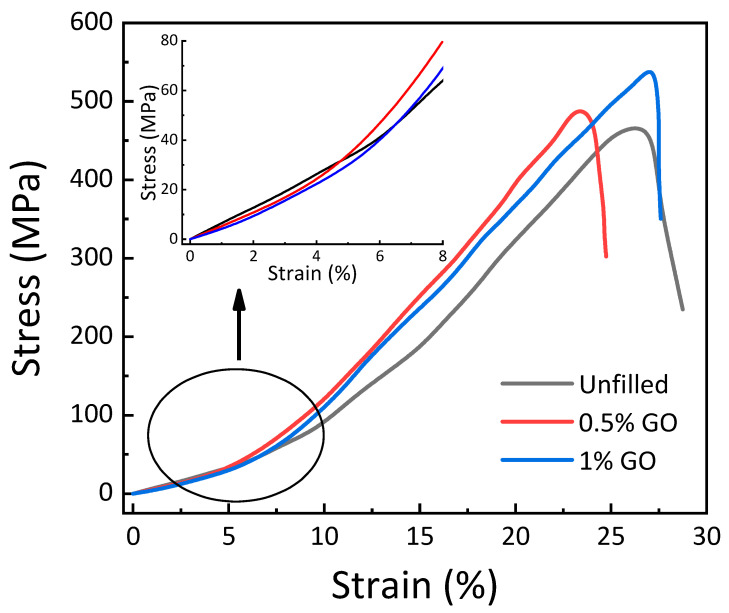
Tensile stress–strain response of GE composite with various GO concentrations.

**Figure 3:**
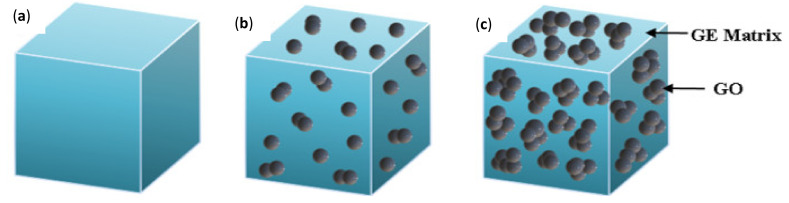
Schematic representation of dispersion of GO particles in the GE matrix: (**a**) GE matrix without filler, (**b**) 0.5 wt. % GO, and (**c**) 1 wt. % GO.

**Figure 4:**
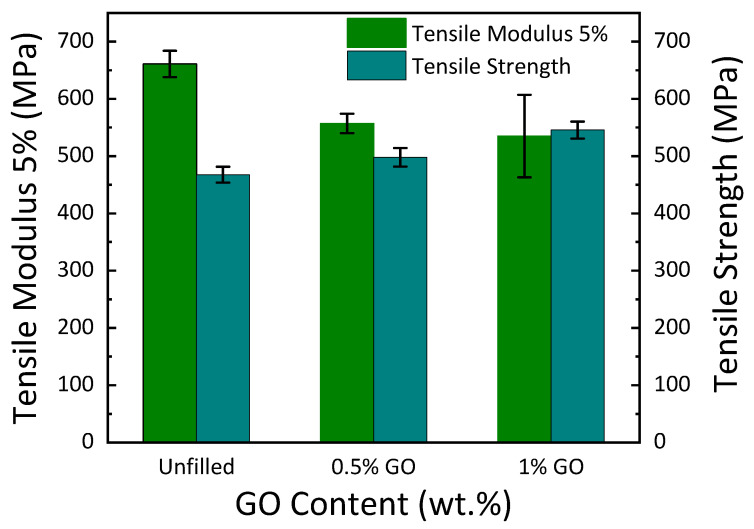
Tensile modulus and tensile strength of polymer composite with varying GO concentration.

**Figure 5:**
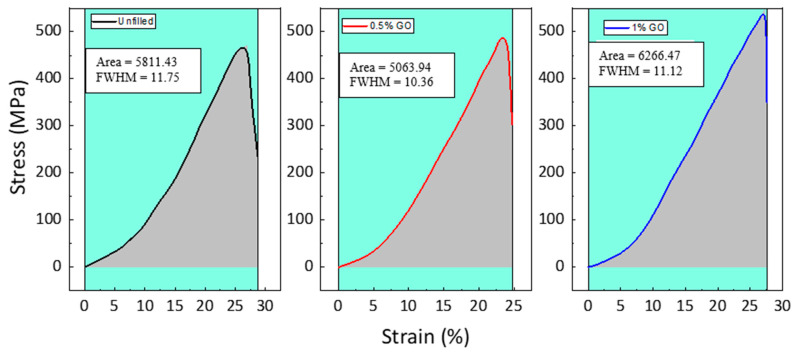
Computation of toughness using the FWHM method for various GO concentrations.

**Figure 6:**
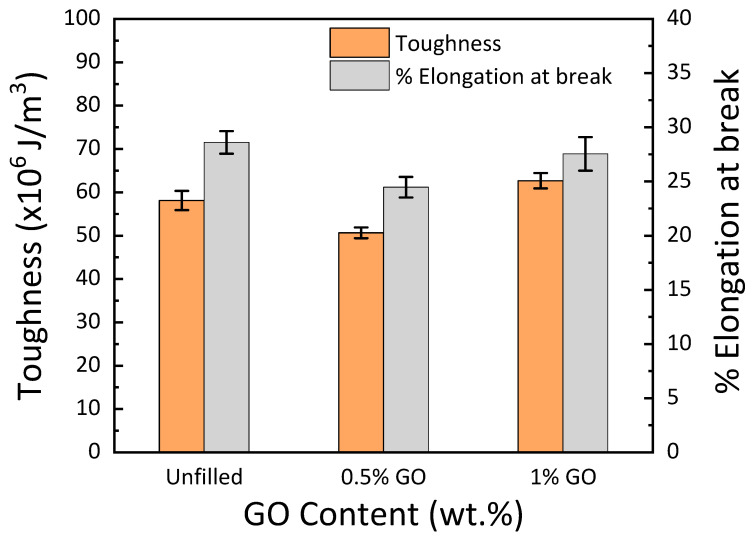
Toughness and % elongation at break of polymer composite with varying GO concentration.

Figure 2 illustrates the effect of GO on the tensile stress–strain behavior of GE composites at ambient temperature. The dispersion of different amounts of GO in the GE matrix is illustrated in Figure 3. The tensile properties, such as strength, modulus, toughness, and percentage elongation at the break of the materials, are reported in Figure 4, Figure 5 and Figure 6. The tensile modulus was reduced by 15.7% with the addition of 0.5 wt. % GO and by 19% with the addition of 1 wt. % GO [see Figure 4]. The tensile modulus results correlate with the storage modulus, which reduces due to the addition of GO. The tensile strength was enhanced by 22.7% with the addition of 0.5 wt. % GO and by 16.7% with the addition of 1 wt. %GO compared to the unfilled GE composite. The higher strength of 0.5 wt. % and 1 wt. % GO encapsulated GE composite could be attributed to the mutual implication of two mechanisms: (i) improved interfacial union at the modified matrix–fiber interface due to enhanced wettability and (ii) improved strength of the matrix due to alteration by GO [15].

The successful transmission of stress from the epoxy matrix to GO along the GO–epoxy contact is responsible for matrix reinforcement. To maximize the application of the effective tensile strength, the load between the matrix to GO should be transferred to the highest potential of the polymer composite. Improving the performance of GO-enhanced epoxy is based on (i) the total interfacial area of GO–epoxy and (ii) the interfacial bonding strength of GO–epoxy. The interfacial bond strength of the GO–epoxy composite depends on the different types of interactions, namely physical, chemical, and mechanical, between graphene oxide and epoxy. Furthermore, the characteristics of composite laminate are determined by the molecular structure of both GO and epoxy components. Nevertheless, the interfacial area between GO and epoxy significantly affects the mechanical behavior of GE composites incorporated with different GO concentrations. In the polymer composite, GO shows a remarkably high explicit surface area, which finally modulates into an enormously high interfacial area of GO–epoxy. The existence of a comprehensive interfacial area enables the impartation of stress from epoxy to GO and, consequently, results in higher mechanical strength of the modified matrix.

Fillers, such as graphene, exhibit a remarkably large surface area. It should be noted that the total volume change in the interfacial polymer zone is considerable at a low GO (0.5 wt. % in this case) concentration. Figure 3a represents the GE matrix without any filler. From Figure 3b, it is observed that at 0.5 wt. % GO concentration, the GO particles are isolated and dispersed in the glass–epoxy matrix, giving rise to a considerably enormous GO–epoxy interfacial area, which correspondingly renders more effective transmission of stress from the matrix to GO. This is accountable for enhancing the tensile strength of 0.5 and 1 wt. % GO–GE composites compared to the unfilled GE composite. The increment in tensile strength of the composites can be correlated to the two-phase system, as shown in Figure 3, and the reduction in cross-link density refers to Table 1 with the addition of GO [36]. Furthermore, the tensile modulus decreased with the incorporation of 0.5 wt. % and 1 wt. % GO, as shown in Figure 4. This decrease correlates with the decrease in the storage modulus of the GO–GE composite, as shown in Table 1.

The toughness of GO–GE composites was estimated using the area under the stress–strain curve and the full width half maximum (FWHM) method, as shown in Figure 5. Filler distribution is related to the toughness of the composites. The decline in toughness and % elongation at break at 0.5 wt. % GO, Figure 6, may be due to non-homogeneous particle distribution in the GE matrix [36]. This correlates to the high loss modulus observed for 0.5 wt. % GO discussed in Section 3.1 (see Figure 1c, Table 1). The results show that the toughness of the GO–GE composite (Figure 6) was enhanced with an increase in GO content (for 1 wt.% GO) due to high particle interaction and homogeneous dispersion, as shown in Figure 3c.

### 3.3. Wear Test

**Figure 7:**
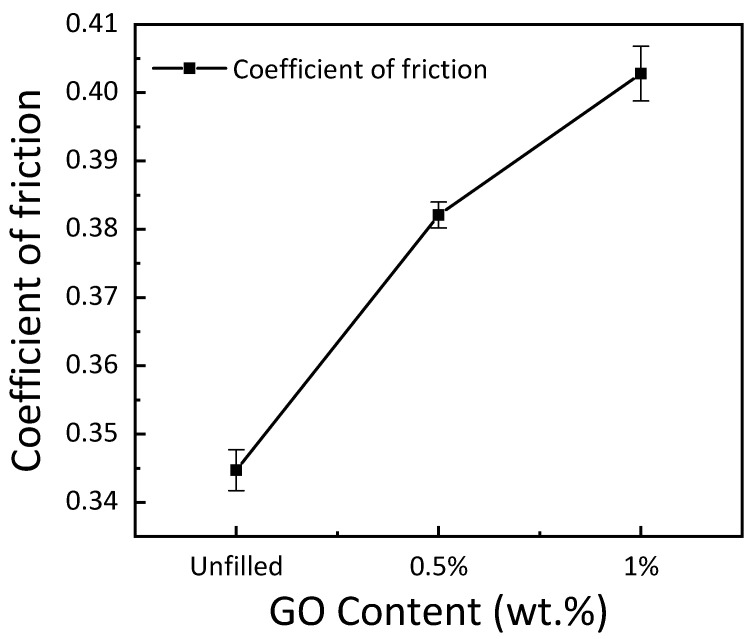
Friction coefficient for varying GO content.

**Figure 8:**
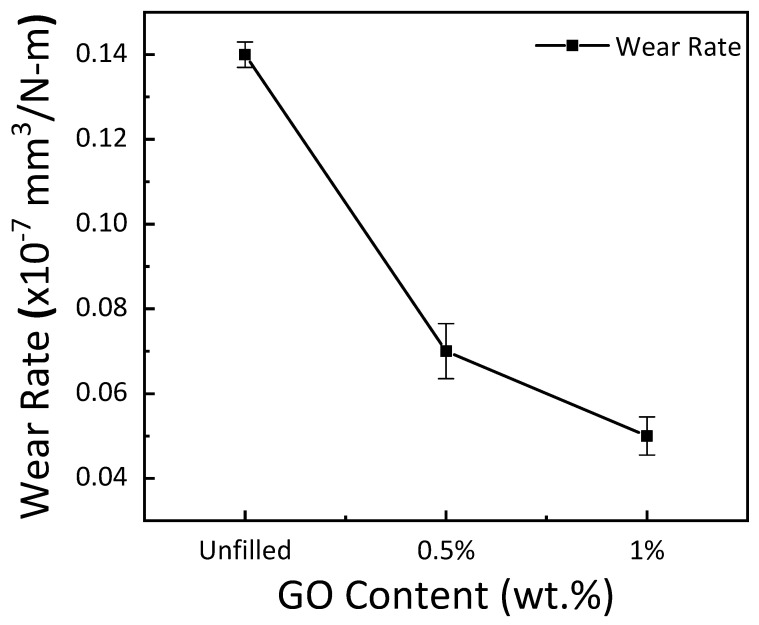
Wear rate for varying GO content.

The coefficient of friction in GE composites as a function of GO concentration is shown in Figure 7. The results show a rise in friction coefficient with an increase in the filler concentration. The growth in the coefficient of friction may be attributed to the GO particle concentration, which is expelled during the sliding test from the epoxy surface [37]. The composites’ wear rate decreased with an increase in GO concentration, as shown in Figure 8. Particle concentration had a considerable impact on the design of the composites as it influenced the wear rate. There was a sharp decrease in wear rate with the inclusion of 0.5 wt. % and 1 wt. % GO in the GE matrix. A 50% and 64.3% reduction in wear rate was reported with the inclusion of 0.5 wt. % and 1 wt. % GO, respectively.

The composite’s dissipated energy was calculated using Archard’s model [38] given by:

(2) $$V=k\frac{SN}{H},$$

where S refers to the sliding distance, N represents the normal load, k is the dimensionless wear constant, and H represents the hardness of the softer material. The loss in volume is directly proportional to the amount of work carried out by the frictional force in the material [38]. The coulomb law of friction is represented by Equation (3):

(3) F=μN,

where F is the friction force, N is the normal load, and μ is the coefficient of friction. The normal force is the load applied during the pin-on-disc experiment substituted in Newtons. *F* is the frictional force obtained from the experiment. Using the Coulomb law of friction (Equation (3)) and Archard’s model (Equation (2)), a relation pertaining to the friction force and wear volume, as represented in Equation (4), was obtained. This corresponds to the dissipation of energy in the system.

(4) $$V=FS\left(\frac{k}{\mu H}\right).$$

**Figure 9:**
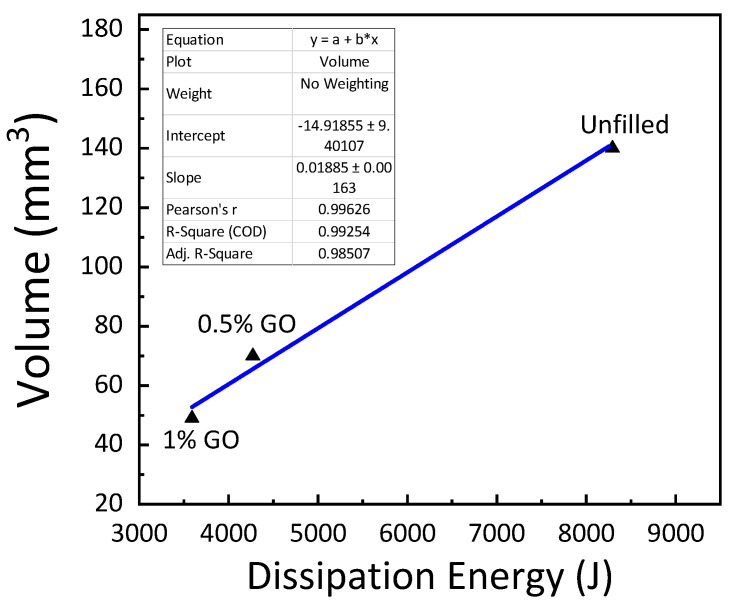
Dissipation energy for varying GO content.

Amongst the investigated composites, the dissipation energy for the 1 wt. % GO–GE composite was significantly low, as shown in Figure 9. As the concentration of GO particles was increased, graphene reacted with the epoxy group and formed functional graphene leading to a reduction in wear rate. By inhibiting graphene from stacking, the amino group of epoxy resin aided in the functionalization process. This resulted in a composite epoxy–graphene structure that was evenly distributed [31]. Consequently, the glass–GO–epoxy composite was competent in transmitting the load across the contact area equally, resulting in a decrease in wear rate. This facilitated the better dispersion of the graphene–epoxy composite structure (Figure 3). A high amount of decrement corresponding to 48.5% and 56.7% reduction in dissipation energy was observed with the inclusion of 0.5 wt. % and 1 wt.% GO. Thus, the composite structure was competent in transferring the load equally through the contact area and consequently led to a reduction in wear rate. The dissipation energy of the system may be directly related to tribological properties such as the friction coefficient and wear rate. The modification in the structure of the polymer composite occurred due to the inclusion of GO in the GE composites, which resulted in the decrease in wear rate. This modification can be primarily accredited to the homogeneous dispersion of GO nanoparticles in the GE matrix (see Figure 3). From the results obtained, it is evident that the inclusion of GO in the GE matrix has a considerable effect on the surface behavior and mechanical characteristics of the composite. The study was limited to the addition of 1 wt. % GO oxide due to agglomeration and the reduction in fracture properties of the polymer composites.

### 3.4. Modeling the Effect of Graphene Oxide Loading on the Stress–Strain Response

In view of capturing the impact of graphene oxide loading on the macroscopic stress–strain behavior of glass–epoxy composites, a simple classical damage mechanics-based model was adopted. In this context, the stress–strain relationship is provided by the following equation [39,40,41,42,43].

(5) σ=Eε(1−d),

where d represents the damage factor and E is the tensile elastic modulus. Subsequently, a Weibull function is used to describe the damage in fiber-reinforced particulate composites provided by:

(6) $$d=I\left(E\varepsilon \right)=1-\exp\left[-{\left(\frac{E\varepsilon }{\sigma_{o}}\right)}^{\beta }\right],$$

where I represents the cumulative probability of failure, β represents the Weibull shape parameter, and *σ_o_* is the Weibull scale parameter. Higher values of $\sigma_{o}$ correspond to higher strength in the material, and lower values of β correspond to a high degree of scattering in the performance of the material. Substituting Equation (6) into Equation (5), we obtain the subsequent stress–strain relationship:

(7) $$\sigma =E\varepsilon \;\exp\left[-{\left(\frac{E\varepsilon }{\sigma_{o}}\right)}^{\beta }\right].$$

By applying the natural logarithm twice to Equation (7), we obtain:

(8) $$\ln\left[\ln\left(\frac{E\varepsilon }{\sigma }\right)\right]=\beta \ln\left(E\varepsilon \right)-\beta \ln\left(\sigma_{o}\right).$$

**Figure 10:**
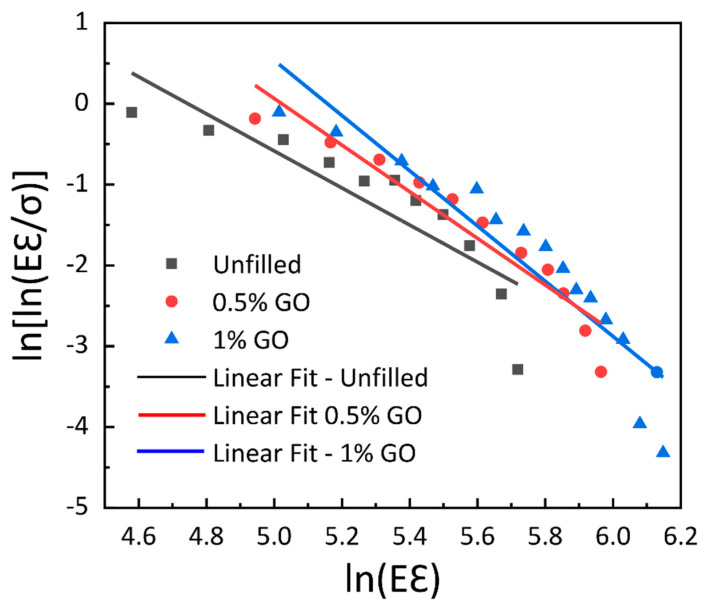
Weibull plots of experimental data for unfilled and filled GE composites.

Equation (8) signifies the equation of a straight line which is determined by the Weibull coordinate system. The measurement of the slope and intercept of the straight line determines the Weibull parameters β and $\sigma_{o}$. The Weibull graphs of experimental data for unfilled and filled GE composite are shown in Figure 10.

**Figure 11:**
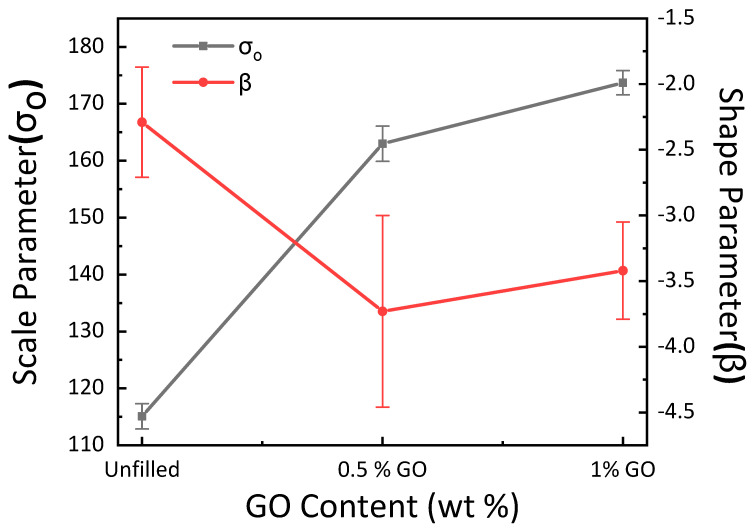
Variation of Weibull parameters as a function of GO content.

From Figure 10, it is observed that the linear curve fitting of the Weibull model is not possible for the complete set of experimental results. It should be noted that for constitutive modeling the tensile modulus is computed at 20% strain in order to cover a larger spectrum. For the higher values of ln (*Eε*), the linear curve does not fit with the experimental results. As a result, the shape and scale parameters of the GE composite vary as a function of GO content, as shown in Figure 11. The critical examination of Figure 11 shows that the scale parameter *σ_o_* manifests a comparable tendency to that of tensile behavior with increasing GO content. On the other hand, there is a sharp decrease in shape parameters with the inclusion of 0.5 wt. % of GO, and a slight increase is observed with the further inclusion of GO content. This correlates with the trend in toughness and % elongation at break, as observed in Figure 6. The lower value of *β* corresponds to a large degree of scattering in the behavior of the material, which is observed with the large standard deviation in Figure 11.

**Figure 12:**
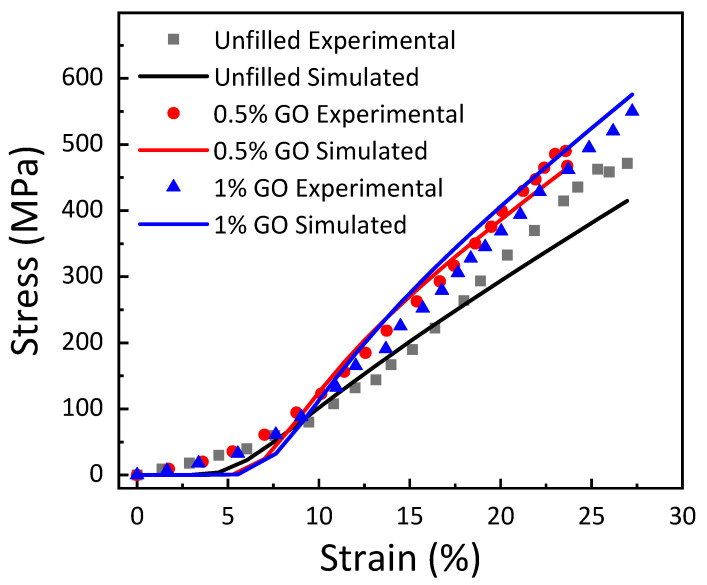
Comparison of experimental and simulated results for unfilled and filled GE composites.

A comparable trend of shape parameters was observed for carbon nanotubes embedded in GE composites due to the variable carbon nanotube content [44]. Similarly, it has been described that a reduction in the value of the shape parameter was observed due to carbon nanotube addition into the carbon–epoxy composite [40]. Nevertheless, with the increase in carbon nanofiber loading, an increasing trend of shape parameters and, therefore, a decline in the degree of scattering in the strength was monitored for carbon nanofiber implanted GE composites [39]. The simulated results were plotted using the values of the shape and scale parameters, and the experimental findings were contrasted with the results, as shown in Figure 12. It can be observed that there is a large amount of variation in the simulated and experimental results, which is due to the large scatter in the shape parameter.

### 3.5. Fractography

**Figure 13:**
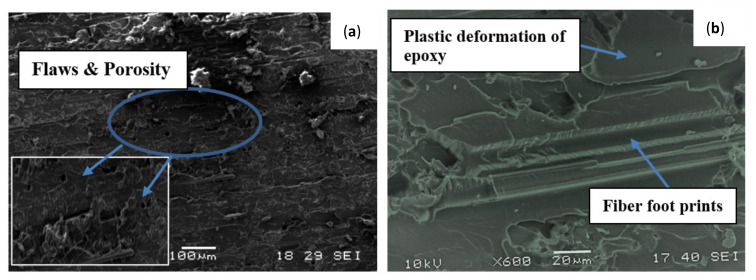
SEM micrographs for tensile test fractured specimen (**a**) 0.5% GO (**left**) and (**b**) 1% GO (**right**) GE composites.

Analysis of the failure mechanisms in the GO-filled glass–epoxy composite using SEM images of tensile fracture surfaces is shown in Figure 13. An uneven and rough fracture surface with significant defects, such as flaws and porosity in 0.5 wt. % GO-filled composite, is observed in Figure 13a, which indicates a brittle fracture and leads to lower tensile properties. This nature of fracture morphology agrees with the experimental results, as displayed in Figure 12. By adding 1 wt. % GO in epoxy, large plastic deformation of the matrix with fiber imprints and smooth fracture surface was observed in Figure 13b. Therefore, the addition of 1 wt. % GO improved the tensile strength of the composite with respect to the 0.5 wt. % GO addition in epoxy. This enhancement of tensile strength is in accordance with the tensile test results, as depicted in Figure 12.

## 4. Conclusions

The effect of various GO concentrations on the macroscopic mechanical behavior of GE composites was investigated. DMA analysis showed a shift in *T_g_* with the increase in GO composition. The cross-link density of the polymer decreased sharply with the addition of 0.5 wt. % GO, whereas at 1 wt. % GO, the amount of reduction in cross-link density was comparable to 0.5 wt. % GO. The rise in the weight percentage of GO, the polymer’s storage, and tensile modulus displayed a similar decreasing trend. The strength of the composite increased with the addition of GO, and was verified with morphological studies using SEM images. The dissipation energy and wear rate decreased significantly with the increase in GO concentration. Furthermore, in this study, the tensile modulus, Weibull shape parameter, and Weibull scale parameter were described to develop a constitutive model of nonlinear stress–strain behavior of unfilled and filled GE composites. Overall, the Weibull scale and shape parameters were expressed as linear weight functions of GO content. Large scatter in the Weibull shape parameter resulted in a large variation in experimental and simulated results. The structure of the composite was found to be dependent on GO concentration. The one-dimensional model can be used as a preliminary tool to assess stress–strain properties prior to experimental testing. The GO-filled glass epoxy composite can be used where the material is exposed under elevated temperature with mechanical loading and to retain the dimension and integrity of structural components at elevated temperatures.

## Figures and Tables

**Table 1:** DMA parameters and cross-link densities of unfilled and GO-filled GE Composites.

Sample	*T_g_* (°C)	Storage Modulus (MPa)	Loss Modulus (MPa)	Cross-Link Density (×10^−2^ mol/cm^3^)
GE–Unfilled	82	1451.72	662.93	3.22
0.5% GO	77	681.08	666.94	1.96
1% GO	72	427.49	413.63	1.08

## Data Availability

Not applicable.
